# Model-Free Adaptive Model Predictive Control for Trajectory Tracking of Autonomous Mining Trucks

**DOI:** 10.3390/s25206434

**Published:** 2025-10-17

**Authors:** Feixiang Xu, Qiuyang Zhang, Junkang Feng, Chen Zhou

**Affiliations:** 1 State Key Laboratory for Fine Exploration and Intelligent Development of Coal Resources, China University of Mining and Technology, Xuzhou 221116, China; xufx92@cumt.edu.cn; 2 Institute of Electronic and Information Engineering, University of Electronic Science and Technology of China, Dongguan 523808, China; 3 School of Information and Control Engineering, China University of Mining and Technology, Xuzhou 221116, China; ts23060155p31@cumt.edu.cn (Q.Z.); fengjk23@cumt.edu.cn (J.F.)

**Keywords:** autonomous mining truck, trajectory tracking, model predictive control, model-free adaptive control

## Abstract

The trajectory-tracking capability of autonomous mining trucks is critical for accomplishing transportation tasks efficiently. However, due to the diverse road surfaces and rugged terrains in open-pit mines, the existing vehicle dynamics models struggle to accurately capture the complex tire–ground interactions. As a result, conventional trajectory-tracking control methods that rely on linear vehicle dynamics models suffer from degraded tracking performance. To this end, this paper proposes a novel trajectory-tracking control framework that integrates model predictive control (MPC) with model-free adaptive control (MFAC). A warm-start strategy is employed to improve the computational efficiency of MPC, while MFAC is utilized to provide real-time compensation for the control deviations generated by MPC during the trajectory-tracking process. To validate the effectiveness of the proposed trajectory-tracking control method, co-simulations were conducted on the CarSim and MATLAB/Simulink platforms under various road conditions and driving scenarios. Simulation results demonstrate that the proposed method can effectively enhance the trajectory-tracking performance of autonomous mining trucks. For instance, under the S-condition with Class E road elevation, the proposed method achieves improvements of approximately 90.83%, 15.05%, and 71.93% in the mean error, maximum error, and root mean square error (RMSE), respectively, compared with the conventional LQR-based trajectory-tracking control method. In addition, the computation time of MPC is only 2 ms, which significantly improves the overall performance of the trajectory-tracking controller.

## 1. Introduction

With rapid development of intelligence in the mining industry, more and more autonomous mining trucks are applied in open-pit mines while the technology is gradually being perfected [1]. As a core component of autonomous driving technology [2], trajectory-tracking technology aims to ensure stable, safe, and precise following of the desired path. And the high-precision nonlinear modeling of autonomous mining trucks is the basis of trajectory-tracking control. However, autonomous mining trucks are subjected to severe disturbances during operation in unstructured operating environments of mine sites [3,4], making it difficult to establish a high-precision, multi-input multi-output nonlinear vehicle model. Moreover, there are also some characteristics that make the modeling of autonomous driving trucks even more difficult, such as the inertial lag effect caused by a heavy body and highly time-varying and intense wheel–ground interactions [5]. Although the existing control strategies perform well on structured roads, their tracking accuracy and robustness performance when dealing with multi-source disturbances still need to be improved [6]. As a result, it is important to find a more effective approach for autonomous mining trucks in open-pit mines.

Existing trajectory-tracking strategies can be mainly divided into two categories: model-based methods [7] and data-driven methods [8]. Model-based trajectory tracking is implemented through a vehicle mechanism model, whose core advantage is that it can be optimized and analyzed according to its explicable physical characteristics. Model-based trajectory tracking calculates control actions to push the predicted trajectory toward the reference. If the model is inaccurate, these actions are calibrated for the wrong results. In the last few years, model-based methods, including sliding mode control (SMC), linear quadratic regulator (LQR), and model predictive control (MPC), have been studied by some researchers. Zhai et al. [9] introduced an MPC-based integrated control method for trajectory tracking and handling stability, improving tracking accuracy across varying road adhesion levels. Recent advances have shown the effectiveness of model predictive control enhanced by orthogonal function approximations. Laguerre-function-based MPC formulations have been successfully applied in power electronic converters [10] and electrical machine drives [11], enabling reduced computational complexity and improved dynamic response. Similar Laguerre-based approaches have also been extended to inverter systems [12]. In parallel, robust and adaptive control strategies have been widely investigated for systems subject to uncertainties and disturbances. Constrained robust adaptive control has been applied to fixed-wing UAVs under parameter variations, while robust fractional-order controllers further improved UAV stability under external disturbances [13]. Adaptive fuzzy sliding mode controllers have also been proposed for robotic manipulators [14], demonstrating superior robustness against modeling errors [15]. For autonomous vehicles, optimization-based predictive controllers have attracted increasing attention. PSO-based linear-parameter-varying MPC was introduced for trajectory tracking [16], showing improved adaptability to nonlinear vehicle dynamics.

It can be found that the studies about trajectory-tracking strategies mentioned above are conducted based on accurate vehicle models. But it is difficult to model autonomous mining trucks directly [17], owing to highly time-varying characteristics caused by the complex dynamic environment. As a result, model-based methods are unsuitable to be applied to trajectory tracking for autonomous mining trucks in open-pit mines.

Data-driven methods avoid the need for explicit vehicle dynamic or kinematic models, which are widely applied in trajectory tracking. As a classic data-driven control method [18], a PID controller based on trial and error was designed by Wael et al. [19], realizing trajectory-tracking accuracy on an expressway. However, the inherent linear characteristic of the PID limits its application in strongly nonlinear and highly coupled operational environments with variable payloads and dynamic disturbances for autonomous mining trucks [20]. Some studies have attempted to learn and summarize experience and patterns from collected data, and then the experience and patterns are incorporated into the control system [18]. Pan et al. [21] proposed a polynomial-based trajectory-fitting method based on past trajectories to design transformation functions, ensuring the planned trajectory’s safety and collision avoidance. However, self-learning relies on lots of offline data and pre-training, and it is confined to fixed scenarios. Moreover, the control performance of self-learning drops sharply under dynamic disturbances or domain shift, limiting application to complex mine-site trajectory tracking [22]. As another scheme of data-driven methods, model-free adaptive control (MFAC) [23] has been widely applied in robotics, UAVs, and vehicle trajectory tracking. MFAC can learn complex time dependencies from training data automatically without manual mathematical modeling and prior knowledge, extracting key temporal features to predict accurately while maintaining prediction stability under input uncertainty. Wang et al. [24] designed a local path-planning algorithm based on a model-free network to forecast surrounding vehicles’ trajectories, which is validated under both lane-change and longitudinal-collision scenarios at low and high speeds. Zhou et al. [25] proposed a model-free finite-time saturation control method with natural constraint, aiming to reduce vehicle vibration and enhance passenger comfort. However, objects of the studies mentioned above are passenger vehicles on a structured pavement, and there are relatively few relevant studies on autonomous mining trucks.

Inspired by previous studies, a trajectory-tracking control strategy that integrates model predictive control (MPC) with model-free adaptive control (MFAC), referred to as MPC-MFAC, is developed to achieve accurate trajectory tracking for autonomous mining trucks. First, to balance modeling accuracy and computational efficiency, a warm-start strategy is introduced to optimize MPC, which preserves the essential dynamic characteristics while significantly improving computational efficiency. Then, a compensation module based on MFAC is designed, which does not rely on the vehicle dynamics model and performs real-time correction of the control signals generated by MPC. In this way, the control deviations caused by modeling inaccuracies are effectively mitigated, thereby enhancing the trajectory-tracking performance of autonomous mining trucks.

The remaining parts of this article are structured as follows: Section 2 introduces the modeling of autonomous mining trucks; Section 3 presents the design of the MPC-MFAC controller; Section 4 is the comparative simulation and results analysis; Section 5 provides the conclusion and future research prospects.

## 2. Vehicle Modeling

Due to the computational complexity arising from high nonlinearity and the requirement for precise parameter identification [26], a simplified representation is required on the basis of accurately characterizing the vehicle dynamics. Accordingly, in this section, a vehicle model formulated in the geodetic inertial coordinate system is established as the foundation for predictive control in the trajectory tracking of autonomous mining trucks.

**Figure 1:**
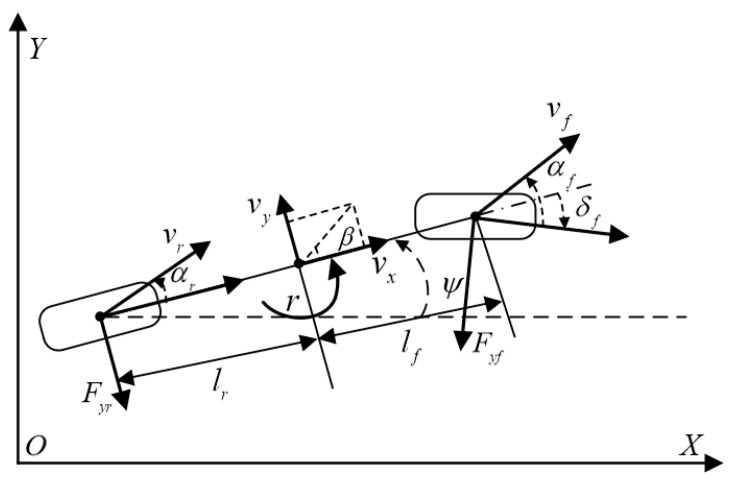
Dynamic model for autonomous mining trucks.

Assuming the mass center coincides with the geometric center of the vehicle o, the lateral tracking error model of an autonomous mining truck is built based on a body-fixed reference frame xoy, as illustrated in Figure 1. We assume that its longitudinal velocity remains unchanged within the predicted time domain. By conducting a force analysis on the vehicle, the vehicle lateral dynamics can be obtained as follows,

(1) $$\begin{array}{l} {\dot{y}}_{o}=v_{x}(\psi +\beta )\\ mv_{x}(\beta +r)=F_{yf}+F_{yr}\\ I_{z}\dot{r}=l_{f}F_{yf}-l_{r}F_{yr} \end{array}$$

where $\left(x_{o},y_{o}\right)$ is the vehicle position in geodetic coordinates, m represents the mass of the vehicle, $v_{x}$ and $v_{y}$ denote the velocity of longitudinal and lateral directions, respectively, β is the sideslip angle of the vehicle, ψ is the yaw angle of the vehicle, r is the yaw rate of the vehicle, $l_{f}$ and $l_{r}$ correspond to the distances from the mass center to the front and rear axles, respectively, $I_{z}$ is the vertical yaw inertia, $F_{yf}$, $F_{yr}$ denote the lateral force on the front or rear axles, respectively.

The front tire slip angle $\alpha_{f}$ and rear tire slip angle $\alpha_{r}$ of the vehicle can be expressed as

(2) $$\begin{array}{l} \alpha_{f}=\beta +\frac{l_{f}}{v_{x}}-\delta_{f}\\ \alpha_{r}=\beta -\frac{l_{r}}{v_{x}} \end{array}$$

As the tire sideslip angle grows, the lateral force exhibits a tendency toward saturation and transitions into the nonlinear domain under extreme operating conditions. For an accurate representation of this characteristic, the magic formula (MF) tire model, a high-fidelity semi-empirical model derived from comprehensive experimental results, is utilized.

(3) $$\begin{array}{c} F_{y}^{MF}(\alpha )=\mu D_{y}\sin(C_{y}\mathrm{acrtan}(B_{y}\alpha_{y}-E_{y}(B_{y}\alpha_{y}-\\ \arctan(B_{y}\alpha_{y}))))+S_{v} \end{array}$$

(4) $$\alpha_{y}=\alpha +S_{h}$$

where μ is the road adhesion coefficient, α represents the tire slip angle, $S_{v}$ and $S_{h}$ stand for the vertical and horizontal drift of the curve, respectively, $B_{y}$ is the stiffness factor, $C_{y}$ is the shape factor of the curve, $D_{y}$ is the peak factor of the curve, representing the maximum value of the curve, $E_{y}$ represents the curvature factor of the curve. They are formulated as

(5) $$\begin{array}{l} C_{y}=f_{Cy1},D_{y}=f_{Dy1}F_{z}^{2}+f_{Dy2}F_{z}\\ B_{y}=f_{By1}\sin[f_{By2}\arctan(f_{By3}F_{z})](1-f_{By4}\left|\gamma \right|)/C_{y}D_{y}\\ E_{y}=f_{Ey1}F_{z}^{2}+f_{Ey2}F_{z}+f_{Ey3}\\ S_{v}=(f_{vy1}F_{z}^{2}+f_{vy2}F_{z})\gamma ,S_{h}=f_{hy1}\gamma \end{array}$$

where γ is the tire camber angle and its value is very small and close to 0. Parameter $f_{cy1},f_{dy1},\ldots ,f_{hy1}$ can be obtained by fitting based on the tire test data. The vertical load of the front axle $F_{zf}$ and rear axle $F_{zr}$ of the vehicle can be estimated by the following formulas,

(6) $$F_{zf}=\frac{mgl_{f}}{l_{f}+l_{r}},F_{zr}=\frac{mgl_{r}}{l_{f}+l_{r}}$$

The nonlinear vehicle dynamics can be expressed as follows,

(7) $$\left\{\begin{array}{l} \dot{x}(t)=f(x(t),\delta_{f}(t)),t\geq 0\\ y(t)=Cx(t) \end{array}\right.$$

where

(8) $$f(x,\delta_{f})=\left[\begin{array}{c} v_{x}\cdot (\psi +\beta )\\ r\\ \;\frac{F_{y}^{MF}(\beta +\frac{l_{f}r}{v_{x}}-\delta_{f})+F_{y}^{MF}(\beta -\frac{l_{r}r}{v_{x}})}{mv_{x}}-r\\ \frac{l_{f}\cdot F_{y}^{MF}(\beta +\frac{l_{f}r}{v_{x}}-\delta_{f})-l_{r}\cdot F_{y}^{MF}(\beta -\frac{l_{r}r}{v_{x}})}{I_{z}} \end{array}\right]$$

## 3. Design of MPC-MFAC

**Figure 2:**
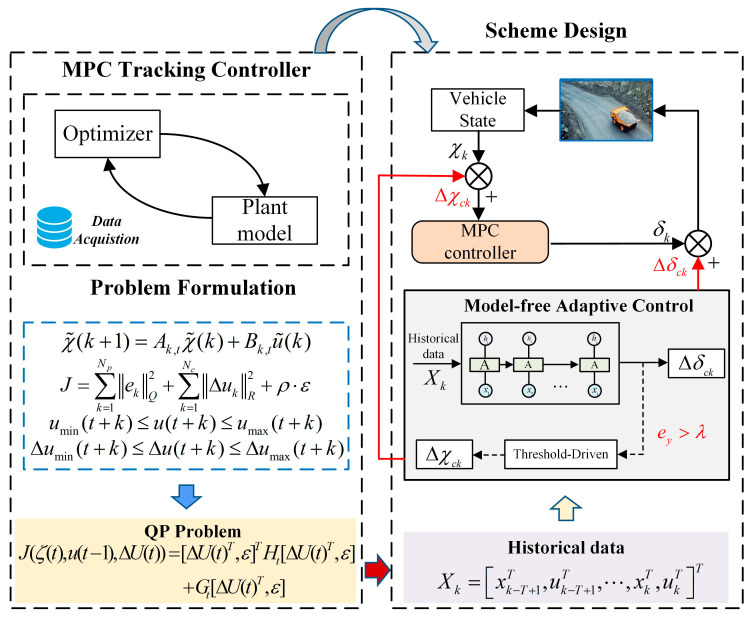
Architecture of the MPC-MFAC for Autonomous Mining Trucks.

An MPC-MFAC tracking control strategy is designed in this paper, whose architecture is illustrated in Figure 2. It can be found that the model-driven and data-driven approaches are hybrid synergized in the architecture of the proposed method, ensuring high-precision trajectory tracking in complex mining environments. Firstly, a basic controller with consideration of vehicle constraints based on MPC is constructed to coordinate optimization across multiple future control horizons. Then the complex trajectory tracking tasks are changed into the QP problem in each sampling interval based on data acquisition. Moreover, the MFAC compensation strategy is introduced to correct discrepancies between actual vehicle trajectories and model-predicted trajectories, balancing control precision and computational efficiency simultaneously.

### 3.1. Construction of MPC Basic Controller

MPC is designed based on a kinematic model to coordinate optimization across multiple future control horizons [27] rather than merely achieve instantaneous error compensation. Furthermore, the optimal control problems are solved through online quadratic programming [28].

To facilitate the application of Equation (7) for MPC design, it can be discretized as follows,

(9) $$\tilde{\chi }\left(k+1\right)=A_{k,t}\tilde{\chi }\left(k\right)+B_{k,t}\tilde{u}(k)$$

where

(10) $$A_{k,t}=\left[\begin{array}{ccc} 1 & 0 & -v_{r}\sin\varphi_{r}T\\ 0 & 1 & v_{r}\cos\varphi_{r}T\\ 0 & 0 & 1 \end{array}\right]\;B_{k,t}=\left[\begin{array}{cc} \cos\varphi_{r}T & 0\\ \sin\varphi_{r}T & 0\\ \frac{\tan\delta_{r}T}{l} & \frac{v_{r}T}{l\cos^{2}\delta } \end{array}\right]$$

where T denotes the sampling period and k represents the discrete time.

At the same time, Equation (9) also can be transformed into a state-space representation:

(11) $$\xi (k|t)=\left[\begin{array}{l} \tilde{\chi }(k|t)\\ \tilde{u}(k-1|t) \end{array}\right]$$

where ξ(k|t) is the state vector at time step k, then a new state space expression is obtained,

(12) $$\begin{array}{l} \xi (k+1|t)={\tilde{A}}_{k,t}\xi (k|t)+{\tilde{B}}_{k,t}\Delta U(k|t)\\ \eta (k|t)={\tilde{C}}_{k,t}\xi (k|t) \end{array}$$

where ${\tilde{A}}_{k,t}=\left[\begin{array}{l} A_{k,t}\\ 0_{m\times n} \end{array}\right.\left.\begin{array}{l} B_{k,t}\\ I_{m} \end{array}\right]$, ${\tilde{B}}_{k,t}=\left[\begin{array}{l} B_{k,t}\\ I_{m} \end{array}\right]$. n represents the dimension of the state quantity and m represents the dimension of the control quantity.

As the key of the MPC, the objective function is designed to ensure rapid, smooth, and precise tracking of the reference trajectory. In this paper, the objective function is designed with explicit consideration of two critical aspects: (1) tracking accuracy: minimization of cumulative tracking errors over the prediction horizon. (2) Stability and ride comfort: limiting the system status and controlling the input and output to change within a reasonable range by considering constraints. As a result, objective functions for MPC are described as follows,

(13) $$J=\sum_{k=1}^{N_{p}}{\left\|e_{k}\right\|}_{Q}^{2}+\sum_{k=1}^{N_{c}}{\left\|\Delta u_{k}\right\|}_{R}^{2}+\rho \cdot \varepsilon$$

where $N_{p}$ is the prediction step size, $N_{c}$ indicates the control step size, $e_{k}$ presents the state-tracking error at time step k, $\Delta u_{k}$ is the increment of input, Q expresses the state weight matrix, R is the weight matrix of input, and ρ signifies the relaxation factor.

To enhance the real-time feasibility of the MPC framework, a warm-starting strategy was adopted. Specifically, the optimal control sequence obtained at the previous sampling instant was used as the initial guess for the current optimization. In addition, recursive feasibility was ensured by shifting the control horizon and appending the terminal input, which reduces solver iterations and improves convergence speed. The initial value of the optimizer can be described as follows,

(14) $$u_{0k}=\{u(k),u(k+1),\ldots ,u(k+N_{p}-2),u(k+N_{p}-1)\}$$

Introducing the following transformation of Equation (12) into Equation (11), the predicted output can be obtained in Equation (14).

(15) $$Y(t)=\Psi_{t}\xi (t|t)+\Theta_{t}\Delta U(t)$$

where

(16) $$Y(t)=\left[\begin{array}{c} \;\eta (t+1|t)\\ \;\eta (t+1|t)\\ \;\eta (t+2|t)\\ \ldots \\ \eta (t+N_{c}|t)\\ \ldots \\ \eta (t+N_{p}|t) \end{array}\right]\;\Psi_{t}=\left[\begin{array}{c} {\tilde{C}}_{t,t}{\tilde{A}}_{t,t}\\ {\tilde{C}}_{t,t}{\tilde{A}}_{t,t}^{2}\\ \ldots \\ {\tilde{C}}_{t,t}{\tilde{A}}_{t,t}^{N_{c}}\\ \ldots \\ {\tilde{C}}_{t,t}{\tilde{A}}_{t,t}^{N_{p}} \end{array}\right]$$

(17) $$\Theta_{t}=\left[\begin{array}{cccc} {\tilde{C}}_{t,t}{\tilde{B}}_{t,t} & 0 & 0 & 0\\ {\tilde{C}}_{t,t}{\tilde{A}}_{t,t}{\tilde{B}}_{t,t} & {\tilde{C}}_{t,t}{\tilde{B}}_{t,t} & 0 & 0\\ \ldots & \ldots & \ddots & \vdots \\ {\tilde{C}}_{t,t}{\tilde{A}}_{t,t}^{N_{c}-1}{\tilde{B}}_{t,t} & {\tilde{C}}_{t,t}{\tilde{A}}_{t,t}^{N_{c}-2}{\tilde{B}}_{t,t} & \ldots & {\tilde{C}}_{t,t}{\tilde{B}}_{t,t}\\ {\tilde{C}}_{t,t}{\tilde{A}}_{t,t}^{N_{c}}{\tilde{B}}_{t,t} & {\tilde{C}}_{t,t}{\tilde{A}}_{t,t}^{N_{c}-1}{\tilde{B}}_{t,t} & \ldots & {\tilde{C}}_{t,t}{\tilde{A}}_{t,t}{\tilde{B}}_{t,t}\\ \vdots & \vdots & \ddots & \vdots \\ {\tilde{C}}_{t,t}{\tilde{A}}_{t,t}^{N_{p}-1}{\tilde{B}}_{t,t} & {\tilde{C}}_{t,t}{\tilde{A}}_{t,t}^{N_{p}-1}{\tilde{B}}_{t,t} & \ldots & {\tilde{C}}_{t,t}{\tilde{A}}_{t,t}^{N_{p}-N_{c}-1}{\tilde{B}}_{t,t} \end{array}\right]$$

(18) $$\Delta U(t)=\left[\begin{array}{c} \Delta u(t|t)\\ \Delta u(t+1|t)\\ \ldots \\ \Delta u(t+N_{c}|t) \end{array}\right]$$

The equality constraints include

(19) $$\begin{array}{c} u(t+k)=u(t+k-1)+\Delta u(t+k)\\ \;\Delta u(t+k)=0 \end{array}$$

where u(t+k) is the control input at time step t+k and Δu(t+k) indicates the control of the increment of the input. The input is forcibly controlled to remain constant from a certain point in time.

To ensure prediction accuracy for subsequent states, inequality constraints for control inputs and their increments on control increments are introduced due to operational characteristics of the mining truck, which are shown as follows,

(20) $$\begin{array}{c} u_{\min}(t+k)\leq u(t+k)\leq u_{\max}(t+k),\\ \Delta u_{\min}(t+k)\leq \Delta u(t+k)\leq \Delta u_{\max}(t+k),\\ k=0,1,\ldots ,N_{c}-1 \end{array}$$

where $u_{\min}$ and $u_{\max}$ are the lower and upper limits of the control quantity, respectively; $\Delta u_{\min}$ and $\Delta u_{\max}$ represent the lower and upper limits for controlling the increment, respectively.

Finally, the optimum is obtained through transforming the objective function into the standard quadratic combined with the constraint conditions, as shown below,

(21) $$J(\zeta (t),u(t-1),\Delta U(t))={\left[\Delta U{\left(t\right)}^{T},\varepsilon \right]}^{T}H_{t}[\Delta U{\left(t\right)}^{T},\varepsilon ]+G_{t}[\Delta U{\left(t\right)}^{T},\varepsilon ]$$

(22) $$H_{t}=\Theta^{T}Q\Theta +R$$

(23) $$G_{t}=2E_{t}^{T}Q\Theta_{t}$$

where $E_{t}$ denotes predicting the tracking error within the time domain.

### 3.2. Design of MPC-MFAC Trajectory-Tracking Controller

This paper proposes an MPC-MFAC control strategy, which not only corrects discrepancies between actual vehicle trajectories and model-predicted trajectories but also provides real-time compensation to the front wheel steering angle commands generated by the MPC solver. The theoretical core of the proposed controller is linear on the controlled object. For a multiple-input multiple-output system, the autonomous mining truck can be expressed as

(24) $$y(k+1)=f(y(k),\ldots ,y(k-n_{y}),u(k),\ldots ,u(k-n_{u}))$$

where f(⋅) is an unknown nonlinear function, $n_{y}$ and $n_{x}$ are two unknown positive integers (the subscripts y and u represent the system output and input, respectively).

**Figure 3:**
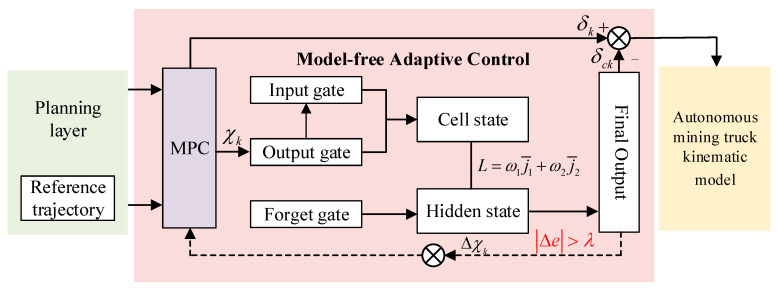
MFAC network architecture.

Based on the high-dimensional data-processing capabilities and relatively simple training process of lightweight long short-term memory (LSTM), MFAC is enhanced to achieve adaptive compensation. The schematic diagram of the MFAC architecture is depicted in Figure 3.

Define the input of the MPC-MFAC network as the historical state-control sequence, and the output is the error compensation amount. The input of the MPC-MFAC network is shown in Equation (25),

(25) $$X_{t}={\left[x_{t-T+1}^{T},u_{t-T+1}^{T},\ldots ,x_{t}^{T},u_{t}^{T}\right]}^{T}$$

where $x_{t}$ is the vehicle state and $u_{t}$ represents the control command.

On one hand, MPC-MFAC can effectively capture long-term dependencies within sequences by introducing the gated mechanisms and cell states of the LSTM network. On the other hand, trajectory-tracking error is transformed into update signals of network parameters through the backpropagation algorithm, enabling the MPC-MFAC to compensate for the errors effectively by learning the mapping relationship.

(26) $$\left\{\begin{array}{l} f_{k}=\sigma (W_{fh}h_{k-1}+W_{fx}X_{k}+b_{f})\\ i_{k}=\sigma (W_{ih}h_{k-1}+W_{ix}X_{k}+b_{i})\\ {\tilde{C}}_{k}=\tanh(W_{\tilde{c}h}h_{k-1}+W_{\tilde{c}x}X_{k}+b_{\tilde{c}}\\ C_{k}=f_{t}\cdot C_{k-1}+i_{k}\cdot {\tilde{C}}_{t}\\ o_{k}=\sigma (W_{oh}h_{k-1}+W_{ox}X_{k}+b_{o}\\ h_{k}=o_{k}\cdot \tanh(C_{k}) \end{array}\right.$$

In Equation (26), σ denotes the sigmoid function, indicating the proportion of retained information. $W_{fh}$, $W_{fx}$, $W_{ih}$, $W_{ix}$, $W_{\tilde{c}h}$, $W_{\tilde{c}x}$, $W_{oh}$ and $W_{ox}$ are the weight matrices, while $b_{f}$, $b_{i}$, $b_{\tilde{c}}$ and $b_{o}$ represent the bias matrices.

To enable MPC-MFAC to learn an effective compensation strategy, the minimization of trajectory-tracking error is defined as the training objective, which is operationalized through a loss function and it is defined as

(27) $$L=\omega_{1}{\bar{j}}_{1}+\omega_{2}{\bar{j}}_{2}$$

where

(28) $$\left\{\begin{array}{l} {\bar{j}}_{1}=E\left[{\left\|e_{t}\right\|}_{2}\right]\\ {\bar{j}}_{2}=E\left[{\left\|\Delta u_{t}-\Delta u_{t-1}\right\|}_{2}\right] \end{array}\right.$$

where ${\bar{j}}_{1}$ and ${\bar{j}}_{2}$ are average tracking error and avoid compensatory fluctuations of the model, respectively. The gradient of the loss function will backpropagate along the network and update the parameters of all gated units in the MFAC, ultimately reducing the prediction error.

To balance control precision and computational efficiency, a threshold-activated MFAC compensation strategy is proposed in this paper. To avoid a heuristic choice of the threshold λ, we first operated the MPC controller without MFAC under representative driving scenarios and collected the lateral error data. The empirical distribution of the absolute lateral error shows that most errors remain small, while larger deviations occur under high-curvature or disturbance conditions. Based on this analysis, we defined the threshold λ as the 95th percentile of the absolute lateral error distribution. Exceeding this value indicates that the MPC-alone performance is likely to deteriorate, and MFAC is then activated for compensation.

Let e(k) denote the set of lateral tracking errors under MPC-only control. The empirical mean and variance are given by

(29) $$\mu_{e}=\frac{1}{N}\sum_{k=1}^{N}e(k),\sigma_{e}^{2}=\frac{1}{N}{\left(e(k)-\mu_{e}\right)}^{2}$$

The threshold is then determined as

(30) $$\lambda =\mu_{e}+\kappa_{e}\sigma_{e}$$

where $\kappa_{e}$ is a tuning factor that specifies the confidence level. In practice, this corresponds to selecting λ near the 95% quantile of the lateral error distribution, ensuring that MFAC is activated only when the MPC steady-state error becomes unacceptable.

(1) Correction for Large Lateral Errors: The MFAC gains experience from historical error sequences and steering commands, suppressing error accumulation effectively, which are represented as

(31) $$\begin{array}{c} x=x_{k}+\Delta x_{ck}\\ y=y_{k}+\Delta y_{ck}\\ yaw=yaw_{k}+\Delta yaw_{ck} \end{array}$$

where $x_{k}$ and $y_{k}$ denote the coordinate of the truck at step k, $yaw_{k}$ represents yaw angle of the truck, $\Delta x_{ck}$, $\Delta y_{ck}$ and $\Delta yaw_{ck}$ stand for the compensation of the inaccurate kinematic model.

(2) Stability Assurance for Small Lateral Errors: Considering that oscillations may be introduced during MFAC compensation due to noise sensitivity and limited training ranges in low-error scenarios, MPC closed-loop control is applied owing to its superior robustness in such cases compared with MFAC compensation. Furthermore, to mitigate errors arising from model inaccuracies in the MPC, the front wheel steering angle should also be compensated in MPC-MFAC. As a result, the output steering angle after compensating can be represented as

(32) $$\delta =\delta_{k}+\Delta \delta_{ck}$$

where $\delta_{k}$ denotes the current front wheel steering angle coordinate of the autonomous mining truck at step k, and $\Delta \delta_{ck}$ represents the compensation of the front wheel steering angle.

## 4. Simulation Results

To validate the trajectory tracking effectiveness of the proposed MPC-MFAC hybrid control strategy, simulations of the autonomous mining truck are conducted with a co-simulation platform based on CarSim 2020.0 and MATLAB/Simulink R2022b. In addition, the vehicle’s control unit is driven by an Intel Core i7-9750 K processor (2.6 GHz) and 8 GB RAM. With warm starting, the average number of solver iterations was reduced by approximately 35%, and the average computation time of MPC decreased from 2.7 to 2 ms. To assess real-time feasibility, we measured the MFAC inference latency on the target platform. The results show that MFAC inference adds a median latency of 5 ms (worst-case 6.2 ms). This improvement ensures that the proposed MPC-MFAC scheme can be executed in real time on embedded hardware.

Parameters of the autonomous mining truck model used in the simulations are detailed in Table 1, while Table 2 and Table 3 shows the configured parameters of the MPC-MFAC. Then comparative simulations under various road conditions and driving scenarios are conducted among the MPC-MFAC, MPC, and LQR approaches.

Subsequently, the vehicle dataset was divided into a training set and a testing set with a ratio of 7:3. The training set was used to train the model, while the testing set was fed into the trained model to evaluate its performance. The final accuracy 97.35% was then calculated based on the prediction results on the testing set.

Considering real-world operational scenarios of autonomous mining trucks, two simulation scenarios are designed under different roads and speeds. One of the scenarios is a double lane-change maneuver, and the speed under this condition is set at 20 km/h and 35 km/h, respectively. To demonstrate the ability of the proposed algorithm to handle both diverse road conditions and significant payload variations of mining trucks, additional comparative experiments were conducted under different loading scenarios. In particular, tests were carried out with varying payloads of the autonomous mining truck so as to further verify the effectiveness and robustness of the method when subjected to large load changes.

### 4.1. Double Lane-Change Maneuver

To further verify the effectiveness of the MPC-MFAC method under both low-speed and high-speed conditions, the obstacle is introduced during straight-line driving. The predefined double lane-change trajectory in this study is set as follows,

(33) $$\begin{array}{ll} Y(X) & =0.36(1+\tanh(0.096(X-80)-1.2)\\ & -0.37(1+\tanh(0.096(X-120)-1.2) \end{array}$$

**Figure 4:**
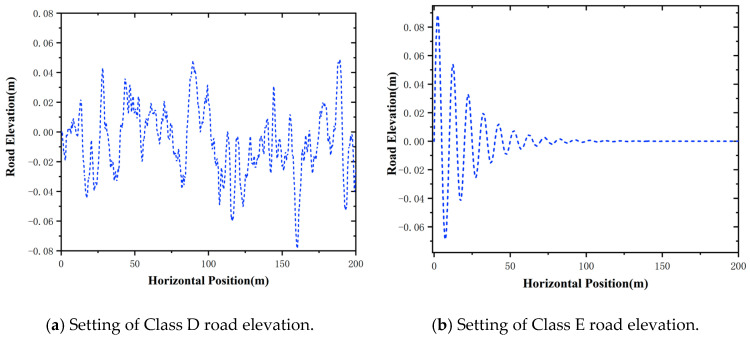
Setting of road elevation.

To further simulate the rugged significant elevation changes and complex geological structure condition in open-pit mines, Class D and Class E road profiles (ISO 8608 standard) are implemented in the simulations. They can reflect the severe unevenness with the road roughness coefficient $G_{d}=16\times 10^{-6}\;m^{3}$ cycle and $G_{d}=256\times 10^{-6}\;m^{3}$ cycle and their road elevation settings are illustrated in Figure 4.

**Figure 5:**
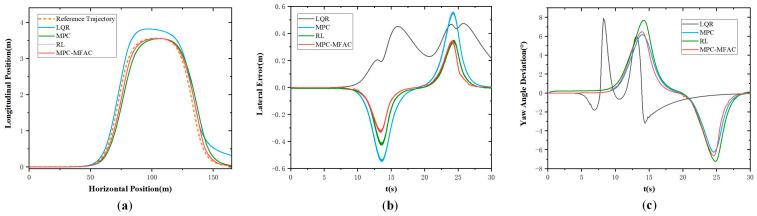
Double lane-change maneuver at the speed of 20 km/h in Class D. (**a**) Actual trajectory. (**b**) Lateral error. (**c**) Yaw angle deviation.

**Figure 6:**
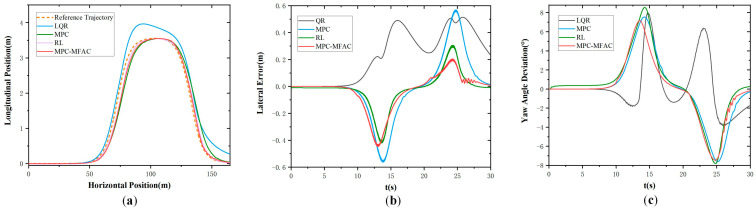
Double lane-change maneuver at the speed of 20 km/h in Class E. (**a**) Actual trajectory. (**b**) Lateral error. (**c**) Yaw angle deviation.

Figure 5 illustrates the simulation results at the speed of 20 km/h at Class D road elevation under the double line-shifting condition. As can be seen from Figure 5, the LQR exhibits big tracking errors and struggles to adapt to complex operating conditions. Meanwhile, MPC demonstrates suboptimal performance in curved path tracking with pronounced lateral deviations. In contrast, the proposed MPC-MFAC, despite exhibiting trajectory fluctuations in error compensation, delivers the highest overall accuracy with the least variability among comparative methods. Figure 6 illustrates the simulation results at the speed of 20 km/h at Class E road elevation under the double line-shifting condition. In contrast, the proposed MPC-MFAC, despite exhibiting trajectory fluctuations in error compensation, achieves the highest overall accuracy among comparative methods when subjected to road conditions and driving scenarios.

**Figure 7:**
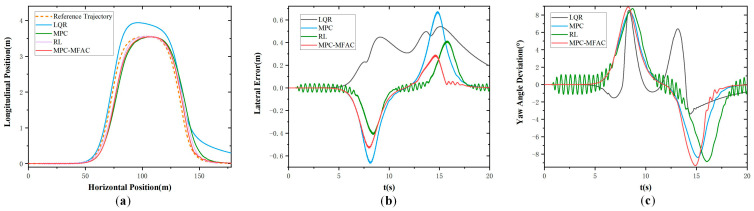
Double lane-change maneuver at the speed of 35 km/h in Class E. (**a**) Actual trajectory. (**b**) Lateral error. (**c**) Yaw angle deviation.

In order to further verify the effectiveness of the proposed strategy under the high-speed driving of autonomous mining trucks, tests were also conducted under the speed of 35 km/h in Class E, whose results are shown in Figure 7. It also can be found that the LQR algorithm exhibits larger tracking errors. And MPC-based path-tracking accuracy is even poorer with larger lateral deviations. However, the proposed MPC-MFAC method experiences minor trajectory oscillations, and its overall precision is the best. Comparative analysis reveals that the trajectory generated by the proposed method is smoother and more closely aligned with the reference trajectory than other methods.

**Figure 8:**
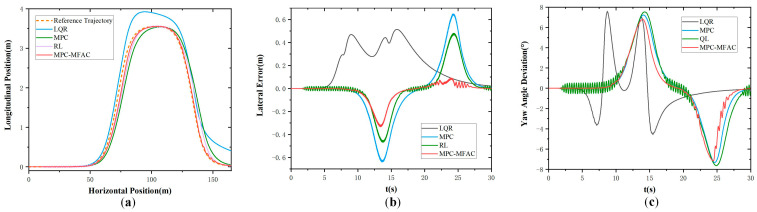
Double lane-change maneuver at the speed of 20 km/h in Class D with a heavy load of 50,000 kg. (**a**) Actual trajectory. (**b**) Lateral error. (**c**) Yaw angle deviation.

In order to further verify the effectiveness of the proposed strategy under large payload variations of mining trucks, comparative tests were conducted on a Class D road profile at a speed of 20 km/h with a heavy load of 50,000 kg, as illustrated in Figure 8. It can be observed that the reinforcement learning (RL)-based controller suffers from severe trajectory oscillations, resulting in poor stability and larger lateral tracking errors. The MPC controller achieves moderate performance but still exhibits noticeable deviations from the reference path under the combined effects of load and road disturbances. In contrast, the proposed MPC-MFAC method maintains the smallest tracking errors and better stability, with smoother trajectories that remain closely aligned with the reference. This comparison further demonstrates the robustness of the proposed framework in handling complex road conditions and significant payload variations.

Table 4 summarizes a quantitative comparison of the four methods. The LQR controller provides acceptable performance under simple road conditions. However, its reliance on linearization results in limited adaptability when facing nonlinear vehicle dynamics. The conventional MPC controller demonstrates improved tracking capability, yet it still suffers from steady-state errors and increased computational demand in complex scenarios. The Q-learning approach shows potential for adaptive decision making, but the lack of model constraints often leads to unstable behavior and slow convergence during training, thereby limiting its applicability in real-time trajectory tracking. In contrast, the proposed MPC-MFAC method effectively combines model predictive control with model-free adaptive control, ensuring both robustness to modeling uncertainties and adaptability to dynamic variations. As a result, MPC-MFAC achieves significantly smaller tracking errors, faster convergence, and more stable control performance across diverse road conditions.

In any type of environment, MPC-MFAC can achieve a smaller lateral tracking error. Specifically, MPC-MFAC reduced the mean by approximately 64.47%, the maximum is reduced by 13.21%, and the RMSE is reduced by 53.90% in Class E at a speed of 20 km/h. At the same time, compared to the MPC controller, MPC-MFAC also exhibited superior performance, achieving reductions of 41.44% for the mean, 21.47% for the maximum, and 39.15% in the RMSE. Similarly, for the speed of 35 km/h under MPC-MFAC, the mean error is reduced by approximately 64.47%, the maximum error by 61.4%, and the RMSE by 1.68% in comparison to LQR. Compared to the QL controller specifically, MPC-MFAC further exhibits superior control accuracy, achieving respective reductions of 0.99% for the mean error, 3.71% for the maximum error, and 0.98% for the RMSE.

In a word, it can be concluded that MPC-MFAC exhibits higher tracking accuracy under this condition, with trajectories characterized by smoother transitions and reduced fluctuations, indicating better adaptability and consistency.

### 4.2. S-Curve Maneuver

The S-curve maneuver is designed to simulate the mining roads characterized by narrow passages and densely clustered bends, which is a common working scenario for trucks. It primarily evaluates the control capability and stability under high-frequency steering and significant lateral acceleration variations. The predefined S-curve trajectory used in the simulations is formulated as follows,

(34) $$Y(X)=\left\{\begin{array}{cc} 0 & x\in [0,15]\\ 3\sin(\frac{\pi (x-15)}{60}) & x\in [15,100]\\ 4\sin(\frac{\pi (x-100)}{60}) & x\in [100,200] \end{array}\right.$$

**Figure 9:**
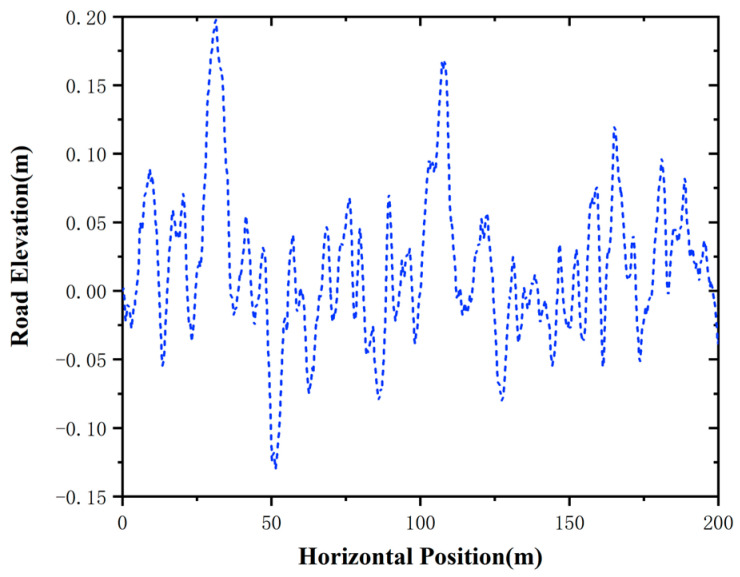
Setting of Class B road elevation.

To further demonstrate the impact of different road conditions and driving scenarios on the controller, Class E and Class B road profiles are implemented in the simulations. They can reflect the severe unevenness with the road roughness coefficient $G_{d}=256\times 10^{-6}\;m^{3}$ cycle and $G_{d}=9.6\times 10^{-6}\;m^{3}$ cycle, and their road elevation settings are illustrated in Figure 9.

**Figure 10:**
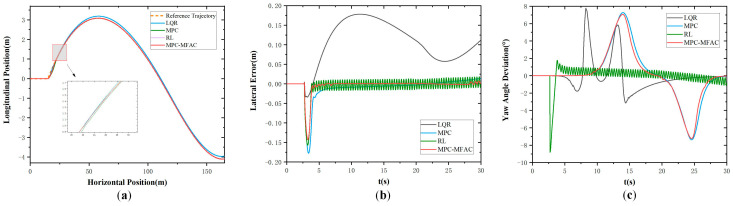
S-Curve Maneuver in Class B at the speed of 20 km/h. (**a**) Actual trajectory. (**b**) Lateral error. (**c**) Yaw angle deviation.

**Figure 11:**
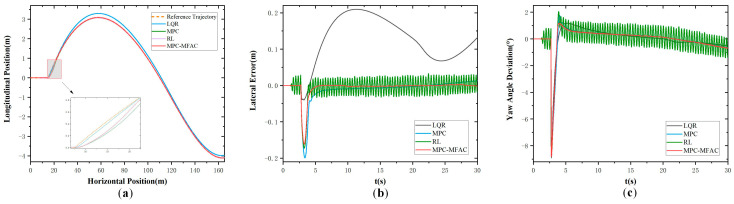
S-Curve Maneuver in Class E at the speed of 20 km/h. (**a**) Actual trajectory. (**b**) Lateral error. (**c**) Yaw angle deviation.

**Figure 12:**
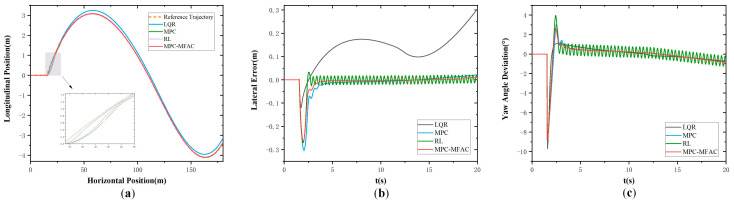
S-Curve Maneuver in Class E at the speed of 35 km/h. (**a**) Actual trajectory. (**b**) Lateral error. (**c**) Yaw angle deviation.

Figure 10 and Figure 11 illustrate simulation results in Class B and Class E at the speed of 20 km/h under the S-curve maneuver condition, respectively. To better simulate the driving environment of unmanned mining trucks and verify the effectiveness of the algorithm, Figure 12 illustrates simulation results in Class E at the speed of 35 km/h. They are the same as the results in the former condition, the LQR generates significant errors during driving, while the MPC produces large errors at curved sections. And the smallest lateral errors with smoother variations in angular error are shown for the MPC-MFA, reflecting the superiority of the proposed method under different conditions. When the unmanned mining truck is operating at high speed and the road surface unevenness is more complex, the MPC-MFAC can still achieve good control performance. Similarly, the detailed trajectory tracking accuracies under this scenario are presented in Table 5.

**Figure 13:**
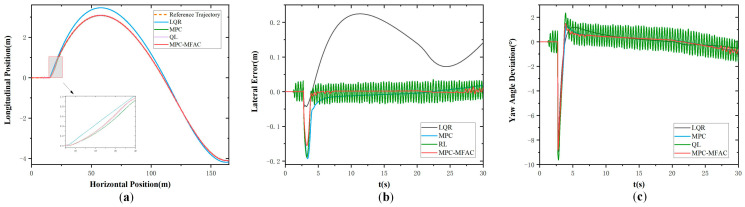
S-Curve Maneuver in Class E at the speed of 20 km/h in Class B with a heavy load of 55,000 kg. (**a**) Actual trajectory. (**b**) Lateral error. (**c**) Yaw angle deviation.

To evaluate the proposed strategy under large payload variations, comparative tests were conducted on a Class B road at 20 km/h with a 55,000 kg load, as shown in Figure 13. The RL-based controller exhibits severe oscillations and large tracking errors, while the MPC controller shows moderate but noticeable deviations under load and road disturbances. In contrast, the proposed MPC-MFAC method achieves smoother trajectories, smaller errors, and superior stability, demonstrating strong robustness to complex road conditions and significant payload changes.

In the comparative experiments, as summarized in Table 5 and illustrated in Figure 10, Figure 11, Figure 12 and Figure 13, the proposed MPC-MFAC method demonstrates clear advantages over the LQR, MPC, and QL controllers. Relative to LQR, MPC-MFAC reduces the root mean square error (RMSE) by 82.81%, reflecting improved adaptability beyond the limitations of linearization. Compared with conventional MPC, MPC-MFAC achieves smaller maximum lateral errors and improved trajectory smoothness, particularly under varying loads and road disturbances. Against the Q-learning controller, MPC-MFAC maintains more stable tracking with reduced oscillations and error dispersion, addressing the instability and slow convergence inherent to reinforcement learning approaches. Overall, the error distributions presented in the box plots indicate that MPC-MFAC delivers the most concentrated results, highlighting its superior robustness, adaptability, and consistency across diverse conditions.

As shown in Table 5, the proposed MPC-MFAC method outperformed LQR across multiple key metrics under 20 km/h conditions. Specifically, MPC-MFAC reduced the mean lateral error by approximately 92.72%, the maximum lateral error by 22.85%, and the RMSE by 82.81% under different speed conditions. When compared to the MPC controller, MPC-MFAC also exhibited superior performance, achieving reductions of 33.07% in the mean lateral error, 18.83% in the maximum lateral error, and 27.24% in the RMSE across varying conditions. Under Class E 35 km/h, the mean error is reduced by approximately 90.84%, the maximum error by 15.05%, and the RMSE by 71.93% based on MPC-MFAC compared to LQR. And it achieves reductions of 57.89% for the mean error, 6.52% for the maximum error, and 29.36% for the RMSE compared to the QL specifically.

Therefore, according to the analysis mentioned above, it can be summarized that MPC-MFAC can eliminate the negative effects of model simplification, improving the stability of vehicles and eliminating the oscillations simultaneously.

## 5. Conclusions

To enhance the trajectory-tracking accuracy of autonomous mining trucks in complex environments, an MPC-MFAC cooperative tracking control strategy is proposed. The main contributions of this study are as follows: (1) to mitigate control deviations in MPC caused by modeling errors, a model-free MFAC approach is introduced to adaptively compensate for the control output; (2) a warm-start strategy is employed to significantly improve the computational efficiency of the MPC controller. Co-simulations were conducted using CarSim and MATLAB/Simulink under both low-speed and high-speed scenarios, including double shift line maneuvers and dense narrow curves. Simulation results demonstrate that, compared to MPC, LQR, and QL, the proposed MPC-MFAC-based trajectory tracking method significantly reduces both lateral deviation and yaw angle deviation. For instance, relative to MPC, the proposed method improves RMSE by 39.15% under the double lane-change condition with Class E road elevation. Then, compared to the LQR, the RMSE reduced by 82.81% under the S-condition with Class E road elevation.

However, there remain several limitations. The current kinematic model does not fully capture axle load transfer or handling stability, and the effects of dynamic obstacles on trajectory tracking are not yet considered. Moreover, validation has so far been limited to simulation. Future work will therefore focus on (1) refining the vehicle dynamics model to improve applicability in high-speed and heavy-load scenarios; (2) incorporating dynamic obstacles into the trajectory-tracking framework and significant parameter variations; (3) extending the MPC-MFAC strategy to hardware-in-the-loop and full-scale autonomous mining truck experiments. By addressing these aspects, the proposed controller can be further strengthened in terms of adaptability and robustness, offering practical value for real-world deployment in tasks such as transportation, terrain exploration, and mineral hauling on uneven mining roads.

## Figures and Tables

**Table 1:** Parameters of autonomous mining truck model.

Parameter/Unit	Value
Total weight 1/kg	31,000
Total weight 2/kg	50,000
Total weight 3/kg	55,000
Distance from the mass center to the front axle/m	2.45
Distance from the mass center to the rear axle/m	2.55
Diameter of the tire/m	1.3
Wheelbase/m	5

**Table 2:** Parameters of MPC-MFAC.

Parameter/Unit	Value
Prediction horizon	100
Control horizon	80
Time step/s	0.1
Weight of lateral error	100
Weight of heading error	70
Relaxation factor	10

**Table 3:** Parameters of LSTM.

Parameter	Value
Batch sizes	1200
Number of input layers	3
Number of hidden layers	6
Output function of sequence classification model	Sigmoid
Output activation function	Tangential hyperbolic
Input gate hidden state weight	6×6
Output gate hidden state weight	6×6

**Table 4:** Trajectory-Tracking Performance under Double Lane-Change Condition.

Variable	Control Method	Mean (m)	Maximum (m)	RMSE (m)
Class D 20 km/h 31,000 kg	LQR	0.2099	0.4737	0.2736
	MPC	0.1229	0.5587	0.2090
	RL	0.0794	0.4267	0.1379
	MPC-MFAC	0.0641	0.3426	0.1135
Class E 20 km/h 31,000 kg	LQR	0.2283	0.5146	0.2974
	MPC	0.1385	0.5687	0.2253
	RL	0.0813	0.4504	0.1384
	MPC-MFAC	0.0811	0.4466	0.1371
Class E 35 km/h 31,000 kg	LQR	0.2497	0.5402	0.3128
	MPC	0.1551	0.6723	0.2579
	RL	0.0961	0.4129	0.1687
	MPC-MFAC	0.0956	0.3123	0.1661
Class D 20 km/h 50,000 kg	LQR	0.2162	0.5149	0.2801
	MPC	0.1497	0.6387	0.2487
	QL	0.1017	0.4806	0.1764
	MPC-MFAC	0.0434	0.3322	0.1255

**Table 5:** Trajectory-Tracking Performance under S-Curve Maneuver.

Variable	Control Method	Mean (m)	Maximum (m)	RMSE (m)
Class B 20 km/h 31,000 kg	LQR	0.1016	0.0129	0.0904
	MPC	0.0121	0.1745	0.0290
	QL	0.0114	0.1568	0.0855
	MPC-MFAC	0.0048	0.1472	0.0223
Class E 20 km/h 31,000 kg	LQR	0.1196	0.2096	0.1367
	MPC	0.0130	0.1992	0.0323
	QL	0.0193	0.1724	0.0304
	MPC-MFAC	0.0087	0.1617	0.0235
Class E 35 km/h 31,000 kg	LQR	0.1322	0.3050	0.1482
	MPC	0.0202	0.3026	0.0521
	QL	0.0152	0.2701	0.0355
	MPC-MFAC	0.0121	0.2591	0.0416
Class B 20 km/h 55,000 kg	LQR	0.1281	0.2245	0.1464
	MPC	0.0150	0.1928	0.0325
	QL	0.0210	0.1884	0.0332
	MPC-MFAC	0.0059	0.1556	0.0224

## Data Availability

The data presented in this study are available from the corresponding author upon request.
